# Reconstruction of the spatial and temporal dynamics of hepatitis B virus genotype D in the Americas

**DOI:** 10.1371/journal.pone.0220342

**Published:** 2019-07-25

**Authors:** Natália Spitz, Francisco C. A. Mello, Aline S. Moreira, Carolina S. Gusatti, Regina M. B. Martins, Selma A. Gomes, Gonzalo Bello, Natalia M. Araujo

**Affiliations:** 1 Laboratory of Molecular Virology, Oswaldo Cruz Institute, FIOCRUZ, Rio de Janeiro, Brazil; 2 Laboratory of Viral Hepatitis, Oswaldo Cruz Institute, FIOCRUZ, Rio de Janeiro, Brazil; 3 Laboratory of Functional Genomics and Bioinformatics, Oswaldo Cruz Institute, FIOCRUZ, Rio de Janeiro, Brazil; 4 Centre for Scientific and Technological Development, State Foundation on Medical Production and Research, Porto Alegre, Brazil; 5 Institute of Tropical Pathology and Public Health, Federal University of Goiás, Goiânia, Brazil; 6 Laboratory of AIDS and Molecular Immunology, Oswaldo Cruz Institute, FIOCRUZ, Rio de Janeiro, Brazil; university of campus biomedico, ITALY

## Abstract

Hepatitis B virus (HBV) genotype D (HBV/D) is globally widespread, and ten subgenotypes (D1 to D10) showing distinct geographic distributions have been described to date. The evolutionary history of HBV/D and its subgenotypes, for which few complete genome sequences are available, in the Americas is not well understood. The main objective of the current study was to determine the full-length genomic sequences of HBV/D isolates from Brazil and frequency, origin and spread of HBV/D subgenotypes in the Americas. Complete HBV/D genomes isolated from 39 Brazilian patients infected with subgenotypes D1 (n = 1), D2 (n = 10), D3 (n = 27), and D4 (n = 1) were sequenced and analyzed together with reference sequences using the Bayesian coalescent and phylogeographic framework. A search for HBV/D sequences available in GenBank revealed 209 complete and 926 partial genomes from American countries (Argentina, Brazil, Canada, Chile, Colombia, Cuba, Haiti, Martinique, Mexico, USA and Venezuela), with the major circulating subgenotypes identified as D1 (26%), D2 (17%), D3 (36%), D4 (21%), and D7 (1%) within the continent. The detailed evolutionary history of HBV/D in the Americas was investigated by using different evolutionary time scales. Spatiotemporal reconstruction analyses using short-term substitution rates suggested times of the most recent common ancestor for the American HBV/D subgenotypes coincident with mass migratory movements to Americas during the 19^th^ and 20^th^ centuries. In particular, significant linkages between Argentina and Syria (D1), Brazil and Central/Eastern Europe (D2), USA and India (D2), and Brazil and Southern Europe (D3) were estimated, consistent with historical and epidemiological data.

## Introduction

Despite the availability of an effective vaccine and potent antiviral treatment, hepatitis B virus (HBV) infection remains a major public health issue, affecting 257 million people worldwide [1, 2]. HBV contains a partially double-stranded DNA genome ~3,200 nucleotides (nt) in length. Based on genomic sequence divergence of > 8%, HBV isolates have been classified into eight genotypes (HBV/A to H) [3–7], further to which two putative genotypes, I and J, have been proposed [8, 9]. The significant diversity within specific HBV genotypes has led to further classification into numerous subgenotypes [10, 11]. HBV genotypes and subgenotypes have distinct geographic distributions and are associated with differences in disease progression, response to antiviral therapy, and clinical outcome [12–15]. In particular, HBV/D is distributed worldwide with predominance in Southeastern Europe, the Mediterranean Basin, the Middle East, and the Indian sub-continent [16]. It has the shortest genome (3,182 nt) and is characterized by a 33 nt deletion at the beginning of the pre-S1 region. Ten subgenotypes (D1 to D10) have been described to date [11, 17], with further corrections and novel classifications of HBV/D subgenotypes reported elsewhere [11, 16, 18, 19]. Relative to HBV/A, HBV/D is associated with poorer clinical outcomes for cirrhosis and hepatocellular carcinoma and lower response to interferon alpha [14].

The origin and evolutionary history of HBV are still controversial and a wide range of HBV substitution rates have been estimated depending on the calibration approach used for the calculation [20, 21]. Studies using external calibrations based on human migrations find slower substitution rates [22, 23], while rates estimated using tip-dating analyses of heterochronous HBV sequences tend to be faster [24, 25]. Accordingly, previous reports on the times of origin and divergence of HBV/D and its subgenotypes have suggested different estimates [23, 24, 26–29]. In the Americas, HBV/D is found across the continent [16, 30–35], although the detailed evolutionary history and phylogeography of this genotype have not yet been examined. In addition, few HBV/D complete genome sequences from Brazil are available in the databanks, limiting the contribution of Brazilian isolates to phylogenetic and phylogeographic studies. The main objectives of this study were to determine the full-length genomic sequences of HBV/D isolates from Brazil, examine the proportion and distribution of HBV/D subgenotypes in American countries, and reconstruct the spatial and temporal diversification of HBV/D in the Americas.

## Materials and methods

### Ethics statement

The study protocol was approved by the Brazilian Ethics Committee for Medical Research (CONEP nº 9604/2004) and the Ethics Committee of Oswaldo Cruz Institute (nº 1.358.935), and all patients signed informed consent before participation. All experiments were performed in accordance with the relevant guidelines and regulations.

### Serum samples

Thirty-nine retrospective HBsAg-positive serum samples (from 2003 to 2013) previously characterized as HBV/D-containing strains, collected from different geographical regions of Brazil (North East, n = 3; Central-West, n = 16; South East, n = 4; and South, n = 16), were selected for HBV complete genome amplification.

### Viral DNA extraction and PCR amplification

HBV DNA was extracted from 0.2 mL serum using a High Pure Viral Nucleic Acid kit (Roche Diagnostics, Germany) according to the manufacturer’s instructions. The amplification of HBV complete genome sequences was attempted with a protocol modified from Günther *et al*., 1995 [36] using 4 μL of the nucleic acid template, primers P1 (5’-CCGGAAAGCTTGAGCTCTTCTTTTTCACCTCTGCCTAATCA-3’) and P2 (5’-CCGGAAAGCTTGAGCTCTTCAAAAAGTTGCATGGTGCTGG-3’), and the following PCR profile: denaturation at 94°C for 4 min followed by 10 cycles at 94°C for 40 s, 55°C for 1 min, and 72°C for 3 min; 10 cycles of 94°C for 40 s, 60°C for 1 min, and 72°C for 5 min; 10 cycles of 94°C for 40 s, 62°C for 1 min, and 72°C for 7 min; 10 cycles of 94°C for 40 s, 62°C for 1 min, and 72°C for 9 min; and a final extension step at 72°C for 10 min. The PCR assay was performed using Platinum *Taq* DNA polymerase and supplied reagents (Invitrogen, Carlsbad, CA) in accordance with product instructions.

### Nucleotide sequencing

PCR products were purified using the Wizard SV Gel and PCR Clean-Up System (Promega, Madison, WI). HBV full-length genome sequences were determined via direct sequencing using a BigDye Terminator Kit v3.1 (Applied Biosystems, Foster City, CA), and sequencing reactions analyzed on an ABI3730xl automated sequencer (Applied Biosystems). Inter and intragenotypic recombination were investigated using SimPlot v3.5.1 software [37], the jumping profile hidden Markov model (jpHMM) [38], and RDP, BootScan, MaxChi, and 3Seq methods embedded in the Recombination Detection Program version 4 (RDP4) [39]. The HBV serological subtype was predicted based on the deduced HBsAg amino acid sequence using the HBV Serotyper Tool available at http://hvdr.bioinf.wits.ac.za/serotyper/. Nucleotide sequences obtained during this study were deposited in the GenBank database under accession numbers MH724214–MH724252.

### Phylogenetic analysis

Multiple sequence alignment was performed using the Muscle program with 66 HBV complete genome sequences (39 sequences determined in this study and 27 reference sequences for HBV genotypes A to J) and subsequently subjected to Maximum Likelihood (ML) phylogenetic analysis. The ML phylogenetic tree was inferred with the online version of the PhyML program [40] under the GTR + I + G nucleotide substitution model selected with SMS (Smart Model Selection in PhyML) [41]. A heuristic tree search was performed with the aid of the SPR branch-swapping algorithm and the reliability of phylogenies estimated with the approximate likelihood-ratio test [42] based on a Shimodaira-Hasegawa-like procedure (SH-aLRT).

### HBV/D sequence dataset from the Americas

To identify the HBV/D subgenotypes circulating in the Americas and determine their proportions in individual countries, a search for HBV/D sequences from the continent available in GenBank by December 2018 was performed using the following queries (for each American country):

((Hepatitis B virus) AND genotype D) AND country = "country name"
((Hepatitis B virus) AND genotype D1) AND country = "country name"
((Hepatitis B virus) AND genotype D2) AND country = "country name"
((Hepatitis B virus) AND genotype D3) AND country = "country name"
((Hepatitis B virus) AND genotype D4) AND country = "country name"
((Hepatitis B virus) AND genotype D5) AND country = "country name"
((Hepatitis B virus) AND genotype D6) AND country = "country name"
((Hepatitis B virus) AND genotype D7) AND country = "country name"
((Hepatitis B virus) AND genotype D8) AND country = "country name"
((Hepatitis B virus) AND genotype D9) AND country = "country name"
((Hepatitis B virus) AND genotype D10) AND country = "country name"

Sequences without a specified subgenotype but more than 600 nt in length were submitted for HBV subgenotyping via phylogenetic analysis and added to the dataset.

### Bayesian phylogeographic analyses

A total of 421 full-length genome sequences from HBV/D subgenotypes D1, D2, D3 and D4 available in GenBank in December 2018 with known country of origin and collection date were grouped into four datasets for each subgenotype and selected for phylogeographic analyses (GenBank accession numbers available in S1 Table). In addition, 29 complete genomes sequenced in this study were included in the analyses. The number of sequences specified by subgenotype and location is shown in Table 1. To avoid bias from overrepresented countries, the online tool CD-HIT Suite (http://weizhongli-lab.org/cdhit_suite/cgi-bin/index.cgi?cmd=cd-hit-est) was used to group sequences with high similarity, and only one representative sequence of each group, prioritizing the one with the oldest collection date, maintained in the dataset.

**Table 1:** Number of sequences specified by subgenotype and locations in phylogeographic analyses.

Subgenotype	Location	Number of sequences^a^
D1	Argentina	9
	Brazil	1
	Central and Eastern Europe	4
	Central Asia	5
	Cuba	1
	East Asia	21
	Iran	16
	Lebanon	14
	New Zealand	8
	South Asia	9
	Southern Europe	8
	Syria	18
	Tunisia	13
	Turkey	20
	Western Europe	13
D2	Argentina	6
	Belgium	4
	Brazil	10
	Central and Eastern Europe	20
	Central Asia	3
	Spain	6
	India	10
	Japan	16
	Middle East	15
	New Caledonia	5
	Taiwan	4
	USA	4
D3	Argentina	19
	Belgium	8
	Brazil	17
	Canada	3
	Cuba	1
	East Asia	8
	Estonia	2
	Haiti	2
	Indonesia	5
	Martinique	1
	Middle East	7
	South Asia	12
	Southern Europe	21
	Sudan	2
	Sweden	6
	USA	10
D4	Australia	3
	Brazil	15
	Canada	3
	Cuba	5
	Haiti	3
	India	12
	Martinique	4
	Polynesia	18

^a^Sequences generated in this study (n = 29) and from Genbank (n = 421) (accession numbers available in S1 Table).

In order to investigate the phylogenetic signal of the datasets, we carried out a likelihood mapping analysis of 10,000 random sets of four sequences (quartets) using IQ-tree v1.6.11 software [43]. In addition, the temporal structure of the datasets was assessed by conducting a regression of root-to-tip genetic distances against year of sampling using TempEst v1.5 [44]. The substitution rate (nucleotide substitutions per site per year, s/s/y), time of the most recent common ancestor (tMRCA, years) and spatiotemporal dynamics of dissemination of each HBV/D subgenotype were estimated using a Bayesian Markov Chain Monte Carlo (MCMC) approach implemented in BEAST v1.8.0 [45] along with BEAGLE v2.1 to improve run time [46]. Analyses were performed using the GTR + I + G nucleotide substitution model, the uncorrelated lognormal relaxed molecular clock model [47] and the Bayesian Skyline coalescent tree prior [48]. The time-scale of the Bayesian tree was calibrated using: (i) the substitution rates directly estimated from the sampling datasets of HBV sequences; or (ii) using informative priors based on two previously published substitution rates, 1.18 x 10^−5^ s/s/y (95% HPD interval: 8.04 x 10^−6^–1.51 x 10^−5^ s/s/y) [49] and 3.7 x 10^−6^ s/s/y (95% HPD interval: 2.2 x 10^−6^–5.5 x 10^−6^) [22].

Spatial reconstruction was achieved by applying a reversible discrete Bayesian phylogeographic model [50]. The Bayesian Stochastic Search Variable Selection (BSSVS) model was implemented, which allows a zero diffusion rate with a positive prior probability. Comparison of the posterior and prior probabilities of individual rates being zero provided a formal Bayes Factor (BF) to test the significance of the linkages between locations. Rates yielding BF > 3 were considered well supported and formed the migration pathway. MCMC was run for a sufficient length to ensure convergence and Effective Sample Size (ESS) values > 100. Uncertainty of parameter estimates was assessed after excluding the initial 10% of the run by calculating the 95% Highest Probability Density (HPD) values using TRACER v1.6 program. Maximum clade credibility (MCC) trees were summarized from the posterior distribution of trees with TreeAnnotator and visualized with FigTree v1.4. Significant migration events were analyzed using SPREAD v.1.0.6 [51].

## Results

### Complete genome sequencing and phylogeny

**Fig 1:**
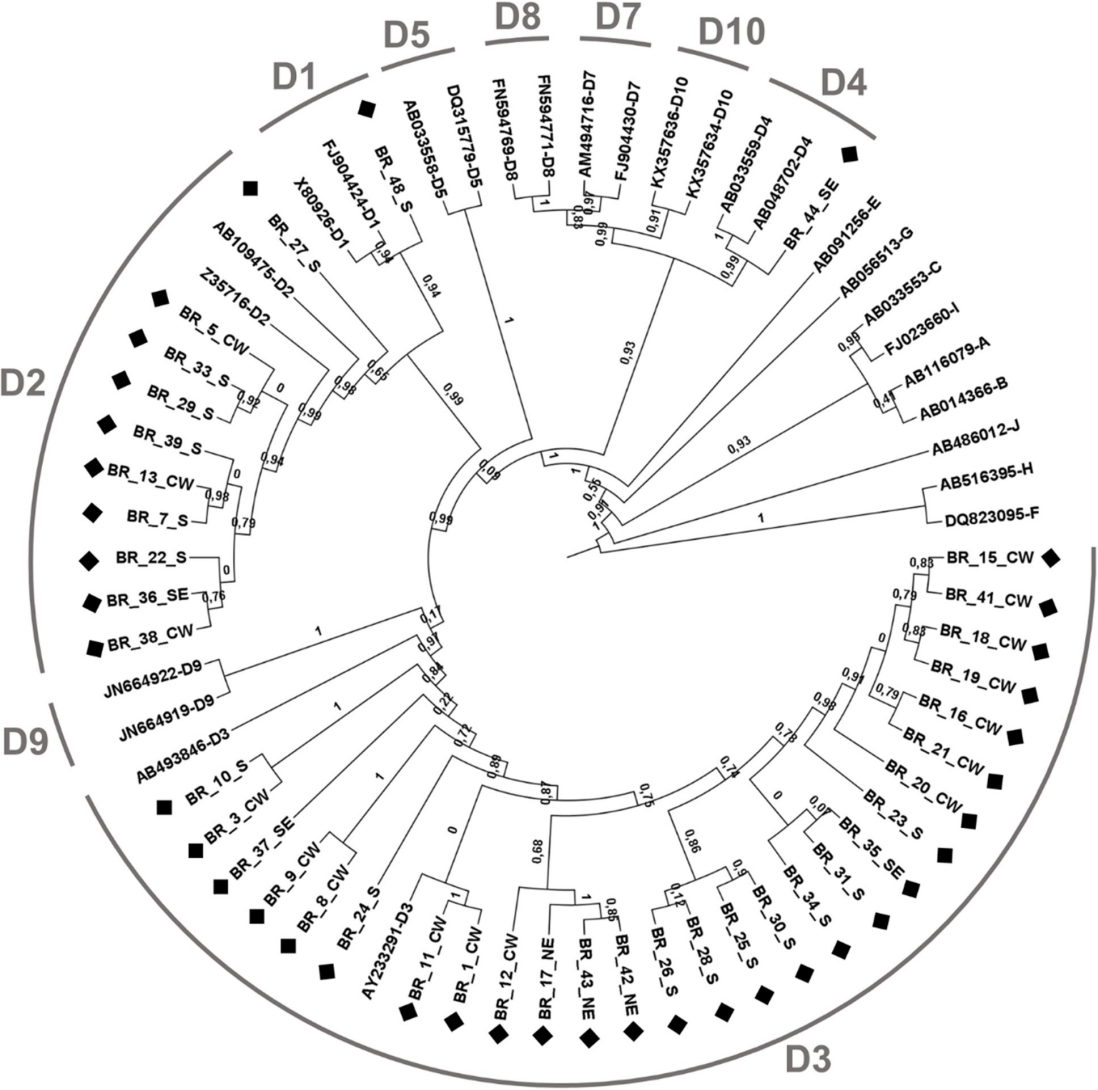
Phylogenetic analysis of HBV complete genome sequences. ML phylogenetic tree of 39 Brazilian HBV/D complete genomes recovered in this study plus 27 reference sequences. The sequences generated are denoted BR, followed by sample number and the geographic region of origin (NE, North East; CW, Central West; SE, South East; S, South), and identified with the symbol ♦. Reference sequences are indicated by their accession number, followed by genotype. The numbers in branches indicate the statistical support (aLRT value).

Thirty-nine full-length HBV/D sequences from different Brazilian regions were successfully amplified and sequenced. All isolates had a genome size of 3,182 nt, with no evidence of in-phase deletion, insertion or recombination. The basal core promoter A1762T/G1764A and precore G1896A mutations were detected in 8/39 (20.5%) and 15/39 (38.5%) sequences, respectively. Fig 1 shows the ML phylogenetic tree of the 39 HBV whole-genome sequences obtained in this study together with 27 reference sequences for HBV genotypes A to J (the bootstrap branch supports are shown in S1 Fig). Based on phylogenetic analysis, HBV genomes were classified as D1 (n = 1), D2 (n = 10), D3 (n = 27), and D4 (n = 1) (Fig 1). Subgenotype D3 sequences were predominantly identified in all the Brazilian geographic regions analyzed (North East, 100%; Central-West, 81%; South East, 50%; South, 56%). The serological subtypes were determined as follows: subgenotype D1 strain was classified as *ayw2*, D2 as 90% *ayw3* and 10% *ayw4*, D3 as 78% *ayw2* and 22% *ayw3*, and the D4 strain as *ayw2*.

### Circulating HBV/D subgenotypes in the Americas

**Fig 2:**
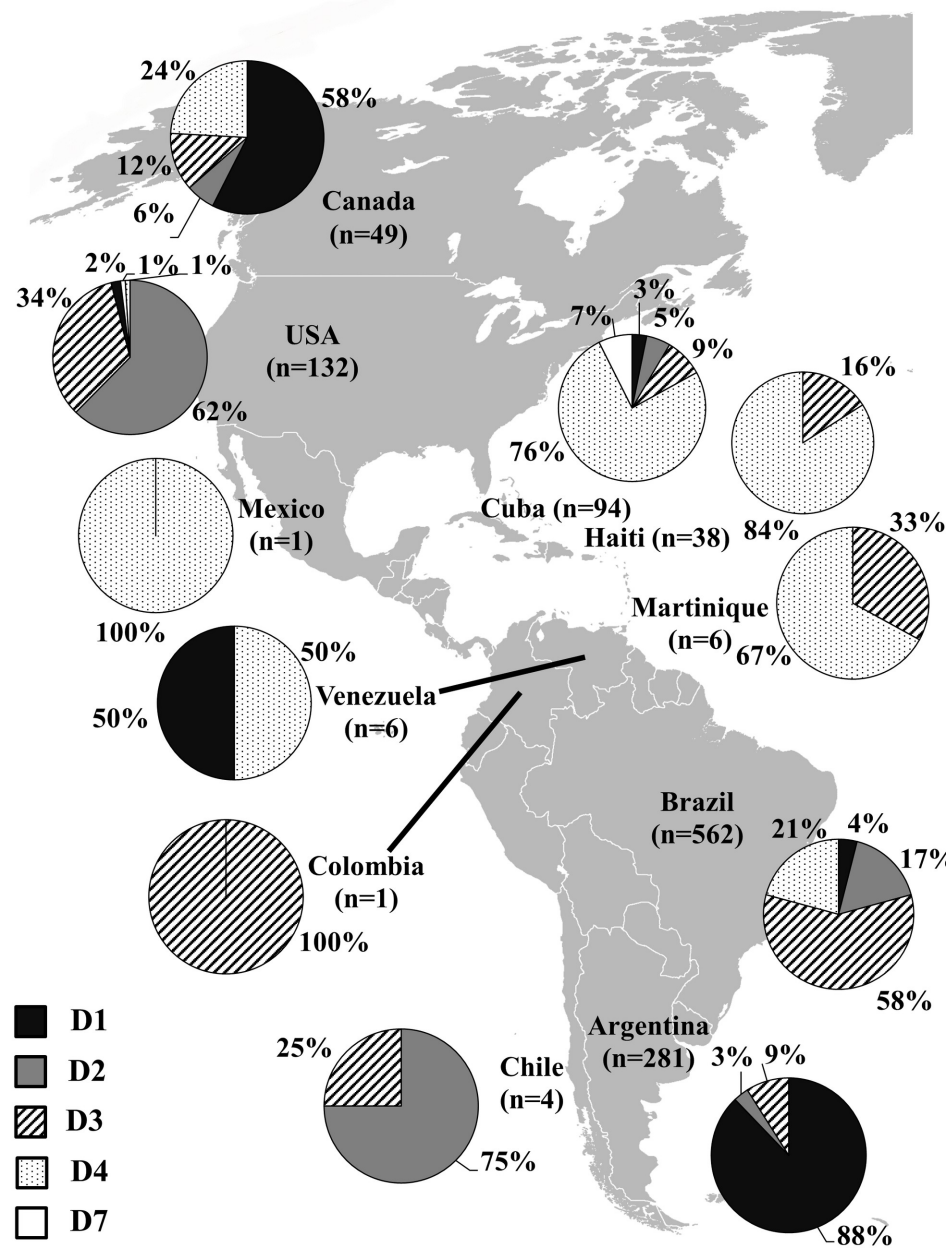
Distribution of HBV/D subgenotypes in the Americas based on 1174 complete and partial genome sequences obtained from GenBank (n = 1135) or generated in this study (n = 39). The map was reconstructed using Wikimedia Commons (https://commons.wikimedia.org/wiki/File:BlankAmericas.png), this figure is similar but not identical to the original image and is therefore for illustrative purposes only.

Using the search queries reported in the Methods section, 1,135 HBV/D sequences isolated in American countries were identified by December 2018 and downloaded from GenBank. Among these, 209 were complete genome and 926 were partial genome sequences. The 39 full-length sequenced genomes were additionally included in the dataset (n = 1,174). HBV/D sequences were obtained from Argentina (n = 281), Brazil (n = 562), Canada (n = 49), Chile (n = 4), Colombia (n = 1), Cuba (n = 94), Haiti (n = 38), Martinique (n = 6), Mexico (n = 1), USA (n = 132) and Venezuela (n = 6). Subgenotypes D1 (26%, 303/1,174), D2 (17%, 196/1,174), D3 (36%, 425/1,174), D4 (21%, 242/1,174) and D7 (1%, 8/1,174) were identified and their distribution patterns throughout the continent presented in Fig 2. Notably, subgenotype D1 was the most prevalent in Argentina (88%, 246/281) and Canada (57%, 28/49), D2 in USA (63%, 83/132), D3 in Brazil, (59%, 331/562), and D4 in Cuba (76%, 71/94) and Haiti (84%, 32/38). The number of HBV/D sequences from other countries was too limited to obtain accurate estimates of subgenotype prevalence (Fig 2).

### Bayesian reconstruction of time-scaled phylogeny and phylogeographic analyses

To estimate the time and epicenter of diversification of the HBV subgenotypes circulating in the Americas, Bayesian MCMC analyses were conducted on 450 (421 from GenBank and 29 from this study) complete genome sequences grouped into four datasets for subgenotypes D1, D2, D3 and D4. Ten D3 genomes sequenced here were excluded from analyses after using the online tool CD-HIT Suite to avoid bias from overrepresentation. Subgenotype D7 was not analyzed due to its recombinant nature (D/E intergenotypic genome) and the small number of available sequences. The phylogenetic signal tested for each dataset showed that the portion of randomly chosen quartets falling in the corners of the likelihood map was >90% (90.8% for HBV/D1, 96.3% for HBV/D2, 95.2% for HBV/D3 and 95.9% for HBV/D4 dataset) (S2A, S2B, S2C and S2D Fig), thus supporting sufficient signal for phylogenetic analysis in all datasets. Moreover, all HBV datasets had a temporal structure as revealed by the positive correlation coefficient between genetic divergence and time (S3 Fig), indicating that the time-scale of HBV can be directly estimated from sampling dates of selected sequences. The estimated mean substitution rates using uninformative priors ranged from 7.17 × 10^−5^ to 3.27 × 10^−4^ s/s/y (Table 2). The coefficient of rate variation for all subgenotypes was significantly higher than zero, supporting the use of a relaxed molecular clock model.

**Table 2:** Bayesian estimates of evolutionary parameters of the HBV/D subgenotypes circulating in the Americas.

Subgenotype	Coefficient of rate variation	Substitution rate^a^	Clade	tMRCA^a^	Location	*PSP*
D1	0.58 (0.50–0.67)	8.41 × 10^−5^ (3.64 × 10^−5^–9.99 × 10^−5^)	root	1667 (1217–1774)	Syria	0.98
			Argentina	1982 (1920–2006)	Syria	0.86
			Brazil	Not determined^b^	Syria	0.68
			Cuba	Not determined^b^	Syria	0.58
D2	0.69 (0.58–0.83)	3.27 × 10^−4^ (1.44 × 10^−4^–5.0 × 10^−4^)	root	1930 (1857–1967)	Central/Eastern Europe	0.56
			Argentina+Brazil	1969 (1941–1984)	Central/Eastern Europe	0.81
			USA	1978 (1935–1981)	India	0.99
D3	0.72 (0.59–0.88)	2.84 × 10^−4^ (4.41 × 10^−6^–5.73 × 10^−4^)	root	1732 (1446–1970)	Southern Europe	0.54
			Argentina	1781 (1589–1979)	Brazil	0.36
			Brazil	1799 (1615–1976)	Southern Europe	0.87
			Canada	1855 (1770–1980)	Brazil	0.87
			Cuba	Not determined^b^	South Asia	0.50
			Haiti	2006 (1999–2006)	Brazil	0.96
			Martinique	Not determined^b^	Brazil	0.99
			USA	1965 (1928–1997)	Belgium	0.57
D4	0.89 (0.66–1.14)	7.17 × 10^−5^ (1.17 × 10^−5^–9.99 × 10^−5^)	root	1614 (283 BCE–1852)	Martinique	0.33
			Brazil	1848 (1062–1946)	Martinique	0.33
			Canada	1890 (1390–1957)	Martinique	0.70
			Cuba	1901 (1402–1969)	Martinique	0.74
			Haiti	1938 (1614–1996)	Martinique	0.71
			Martinique	1912 (1482–1975)	Martinique	0.74

BCE (Before the Current Era)

^a^The 95% HPD interval is displayed in parentheses

^b^Only one sequence available

**Fig 3:**
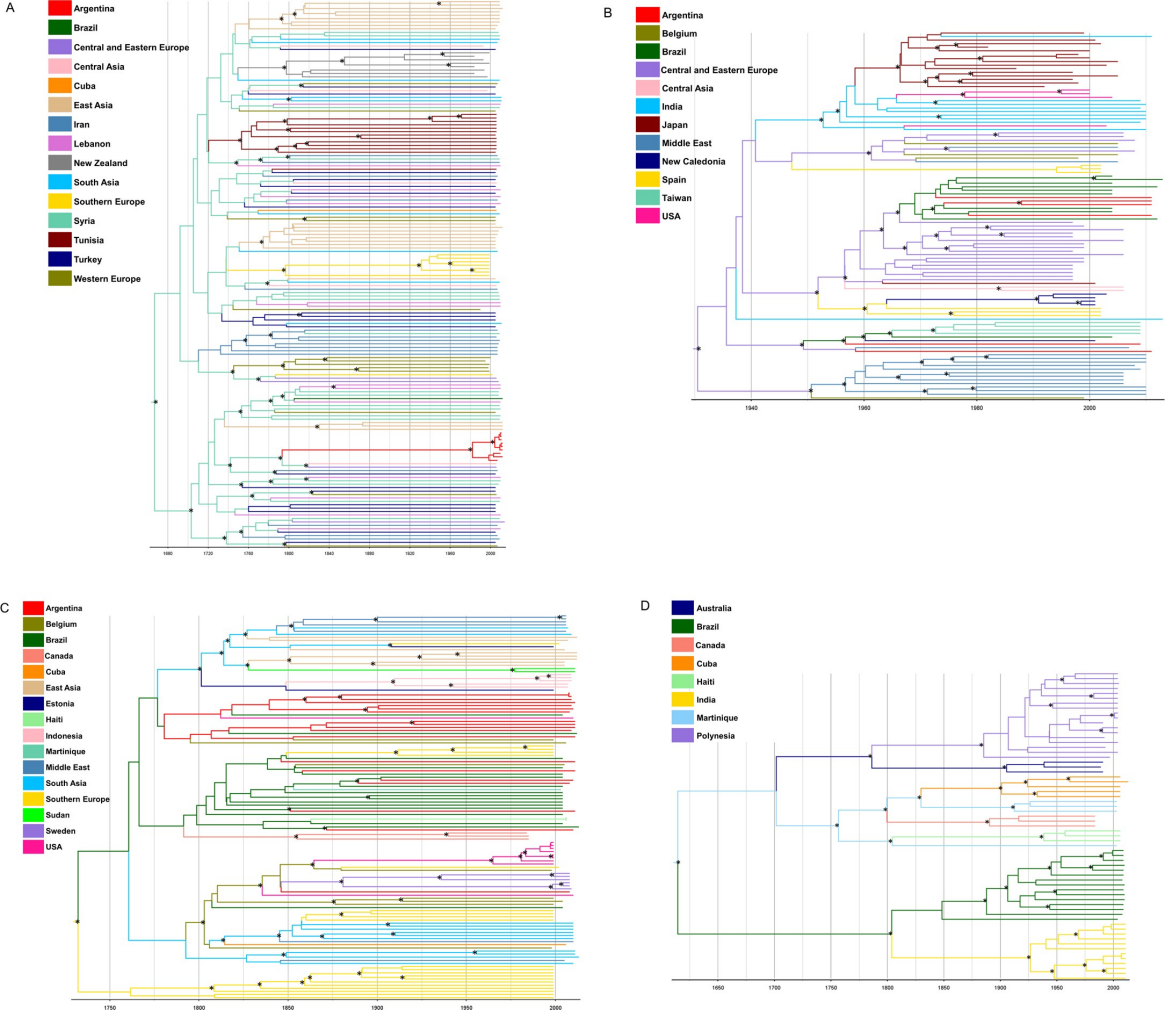
Bayesian maximum clade credibility tree of HBV/D full-length genome sequences. Branches are colored according to the potential locations of the parental node (colored legend in the figure). The scale at the bottom of the tree represents years before the last sampling time. All nodes marked with an asterisk show posterior probability > 0.90. The tree was automatically rooted under the assumption of a relaxed molecular clock. A) Subgenotype D1, last sampling time 2014; B) Subgenotype D2, last sampling time 2013; C) Subgenotype D3, last sampling time 2013; D) Subgenotype D4, last sampling time 2013.

**Fig 4:**
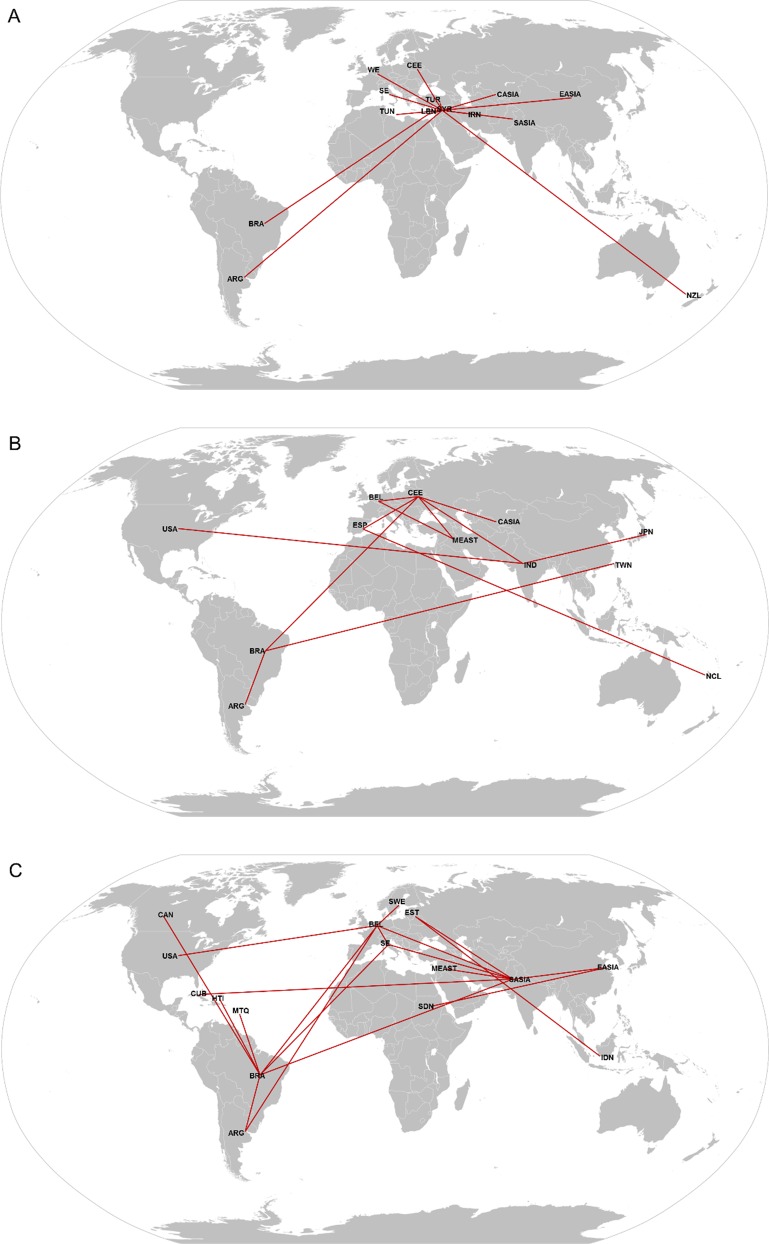
Bayes factor (BF) test for significant non-zero HBV/D migration rates worldwide. Only rates supported by BF greater than 3 are indicated. A) Subgenotype D1 (ARG: Argentina, BRA: Brazil, CASIA: Central Asia, CEE: Central/Eastern Europe, EASIA: East Asia, IRN: Iran, LBN: Lebanon, NZL: New Zealand, SASIA: South Asia, SE: Southern Europe, SYR: Syria, TUN: Tunisia, TUR: Turkey, WE: Western Europe); B) Subgenotype D2 (ARG: Argentina, BEL: Belgium, BRA: Brazil, CASIA: Central Asia, CEE: Central/Eastern Europe, ESP: Spain, IND: India, JPN: Japan, MEAST: Middle East, NCL: New Caledonia, TWN: Taiwan, USA: United States of America); C) Subgenotype D3 (ARG: Argentina, BEL: Belgium, BRA: Brazil, CAN: Canada, CUB: Cuba, EASIA: East Asia, EST: Estonia, HTI: Haiti, IDN: Indonesia, MEAST: Middle East, MTQ: Martinique, SASIA: South Asia, SE: Southern Europe, SDN: Sudan, SWE: Sweden, USA: United States of America). The maps were reconstructed using Wikimedia Commons (https://commons.wikimedia.org/wiki/Maps_of_the_world#/media/File:BlankMap-World.svg), this figure is similar but not identical to the original image and is therefore for illustrative purposes only.

Data from spatiotemporal reconstruction analyses using such substitution rates suggest that subgenotype D1 possibly originated in Syria (posterior state probability (*PSP*) = 0.98). From Syria, D1 migrated to Cuba (*PSP* = 0.58) and Brazil (*PSP* = 0.68), but it was not possible to determine the time of introduction, since only one D1 sequence was available from these countries. In Argentina, the most probable location for the origin of D1 was also Syria (*PSP* = 0.86) and its tMRCA calculated as 1982 (95% HPD: 1920–2006) (Table 2; Fig 3A). Calculation of the BF using SPREAD software revealed significant epidemiological relationships (BF > 3) between Brazil/Lebanon and Argentina/Syria (Fig 4A).

The phylogeography of subgenotype D2 suggests that the most probable epicenter was Central/Eastern Europe (Estonia, Russia, Poland and Serbia) (*PSP* = 0.56). The majority of the Brazilian and Argentine sequences formed a single cluster (posterior probability (*PP*) = 0.99) whose tMRCA was dated back to 1969 (95% HPD: 1941–1984), with Central/Eastern Europe as the most probable source location (*PSP* = 0.81). On the other hand, sequences from USA grouped with Indian sequences, and at least two different introductions of this subgenotype seem to have occurred in USA. The main time of introduction was estimated between 1966 and 1978 from India (*PSP* = 0.99) (Table 2; Fig 3B). BF calculation revealed strong epidemiological relationships between Brazil/Central/Eastern Europe, Brazil/Argentina, and USA/India (Fig 4B).

Phylogeographic analysis showed Southern Europe (Italy and Spain) (*PSP* = 0.54) as the putative origin of subgenotype D3. Viral sequences from Argentina, Brazil and USA segregated into different clusters, suggesting multiple introductions in these countries, with tMRCAs of the main clusters calculated as 1781 (95% HPD: 1589–1979), 1799 (95% HPD: 1615–1976) and 1965 (95% HPD: 1928–1997), respectively. In contrast, Canadian D3 sequences constituted a single monophyletic group whose tMRCA was dated back to 1855 (95% HPD: 1770–1980). Among the Caribbean D3 sequences, Martinique and Haiti clustered together with most sequences from Brazil, while Cuba showed a distinct dispersal pathway, grouping with viral sequences from South Asia (India and Pakistan) (Table 2; Fig 3C). Significant migration events between Argentina/Belgium, Brazil/Argentina, Brazil/Martinique, Brazil/Haiti, Brazil/Southern Europe, Brazil/Belgium, Brazil/Canada, USA/Belgium and Cuba/South Asia were suggested (Fig 4C).

Unexpectedly, spatiotemporal reconstruction of subgenotype D4 highlighted Martinique (*PSP* = 0.33) as the most probable place of origin. All D4 sequences branched in country-specific monophyletic groups that probably arose between the middle 1800s and middle 1900s (Table 2; Fig 3D).

Additionally, we tested alternative evolutionary time scales inferred by using informative priors based on two previously published substitution rates for HBV: (i) 1.18 x 10^−5^ s/s/y (95% HPD interval: 8.04 x 10^−6^–1.51 x 10^−5^ s/s/y) calculated on the basis of heterochronous sampling calibration using ancient sequences [49], and (ii) 3.7 x 10^−6^ s/s/y (95% HPD interval: 2.2 x 10^−6^–5.5 x 10^−6^) estimated by means of external calibrations based on human migrations [22]. As shown in Table 3, time-scale reconstructions under both substitution rates informative priors suggested, in the vast majority of cases, tMRCA estimates for HBV/D subgenotypes in the Americas dating back to the pre-Columbian era (before 1492).

**Table 3:** Time to the most recent common ancestor (tMRCA) estimates for HBV/D subgenotypes by calibration with previously published substitution rates.

Subgenotype	Clade	tMRCA^a^	
		Substitution rate [49] 1.18 x 10^−5^ (8.04 x 10^−6^–1.51 x 10^−5^)	Substitution rate [22] 3.7 x 10^−6^ (2.2 x 10^−6^–5.5 x 10^−6^)
D1	Root	424 BCE (1478 BCE–274)	5535 BCE (10532 BCE–2877 BCE)
	Argentina	1781 (1523–1938)	1296 (361–1802)
	Brazil	Not determined^b^	Not determined^b^
	Cuba	Not determined^b^	Not determined^b^
D2	root	136 BCE (1416 BCE–674)	4614 BCE (9605 BCE–1732 BCE)
	Argentina+Brazil	1001 (500–1324)	1066 BCE (3191 BCE–153)
	USA	1305 (842–1717)	123 BCE (1974 BCE–1227)
D3	root	481 BCE (1710 BCE–346)	5764 BCE (27666 BCE–1870 BCE)
	Argentina	202 (591 BCE–740)	3715 BCE (11408 BCE–962 BCE)
	Brazil	165 (648 BCE–705)	3663 BCE (9718 BCE–1204 BCE)
	Canada	954 (255–1526)	1231 BCE (7535 BCE–1180)
	Cuba	Not determined^b^	Not determined^b^
	Haiti	1993 (1979–2005)	1962 (699 BCE–2006)
	Martinique	Not determined^b^	Not determined^b^
	USA	1688 (1375–1889)	1040 (2624 BCE–1864)
D4	root	336 BCE (2010 BCE–736)	5285 BCE (11485 BCE–1616 BCE)
	Brazil	1097 (507–1497)	840 BCE (3392 BCE–671)
	Canada	1424 (981–1708)	192 (1321 BCE–1236)
	Cuba	1399 (974–1656)	125 (1405 BCE–1114)
	Haiti	1621 (1300–1878)	849 (306 BCE–1548)

BCE (Before the Current Era)

^a^The 95% HPD interval is displayed in parentheses.

^b^Only one sequence available

## Discussion

To our knowledge, the present study is the first to reconstruct the spatial and temporal dynamics of HBV/D in the Americas using a Bayesian framework. To this end, we conducted a survey of HBV/D sequences and assessed the distribution of five among the 10 HBV/D subgenotypes (D1, D2, D3, D4 and D7) throughout the continent (Argentina, Brazil, Canada, Chile, Colombia, Cuba, Haiti, Martinique, Mexico, USA, and Venezuela). Interestingly, Cuba was the only country in which all five subgenotypes were identified, which may be attributable to interactions of the island with different countries over the years [34]. Brazil is the largest country in the Southern Hemisphere corresponding to almost half of the area of South America. In Brazil, HBV/D was identified countrywide [52–54], with the highest rates in the Southern region where an intense flow of European immigrants had occurred [55, 56]. Here, we introduced 39 HBV/D full-length sequences corresponding to 19% (39/209) of the complete HBV genomes from the Americas deposited in GenBank by December 2018. These new genomes represent all HBV/D subgenotypes (D1, D2, D3 and D4) circulating in Brazil as well as the first sequenced Brazilian D1 genome.

Recent findings from two studies using ancient samples pointed out that HBV has been infecting humans for at least 7,000 years [49, 57]. Owing to the lack of agreement concerning the HBV substitution rate, the times of origin and divergence of HBV genotypes and subgenotypes are largely uncertain [21]. Accordingly, the results obtained from the phylodynamics and phylogeography of HBV should always be carefully analyzed in combination with historical and epidemiological knowledge.

In this study, we reconstructed the evolutionary history of HBV/D in the Americas by using substitution rates estimated from the sampling dates of the sequences or by using rates previously estimated for HBV as informative priors for calibration of time-scale. The time-scale reconstructions based on ancient heterochronous sequences [49] and external calibration [22] (long-term substitution rates) recovered much older tMRCAs than those based on substitution rates directly estimated from modern heterochronous sequences (short-term substitution rates). Almost all tMRCAs obtained with long-term substitution rates precede the European discovery of the Americas (~500 years ago), which is incompatible with epidemiological and historical data, since only HBV/F and HBV/H are thought to be genotypes originating in the Americas [3, 23, 58–60]. On the other hand, spatiotemporal reconstruction using short-term substitution rates provided an epidemiologically realistic scenario, suggesting tMRCAs for the American HBV/D subgenotypes coincident with mass migratory movements to Americas during the 19^th^ and 20^th^ centuries. Therefore, our results corroborate the concept that tip-dating analyses of modern heterochronous HBV sequences are probably more appropriate in dating recent dispersal events [21, 22]. Conversely, the use of deep calibrations is likely to be effective to estimate events that are distant in time, such as the origin of viral genotypes/subgenotypes.

Our phylogeographic reconstruction showed that Syria is the most likely location for the origin of subgenotype D1. Syrian D1 sequences were not available when previous studies suggested that this subgenotype originated in Turkey [24, 26]. In fact, Syria and Turkey are geographically close and have historical links that tie the two neighboring countries together. The phylogeographic data suggest that the Cuban D1 originated from Syria. According to Paredes (2000) [61], between the second half of the 19^th^ century and the first half of the 20^th^ century several thousand of people, mainly from Syria, Lebanon, Palestine, Turkey, and Egypt had migrated to Cuba. The introduction of D1 in Brazil was also suggested to have occurred from Syria. This proposal is reinforced by the fact that Brazil has the largest Arab colony outside the Middle East. It is estimated that about 7% of the Brazilian population is of Arab origin, the majority from Syria and Lebanon, and the migratory movement from these countries to Brazil was initially documented from the second half of the 19^th^ century [62, 63]. Phylogeographic analysis of D1 sequences from Argentina suggested a single introduction from Syria during the early 1980s (95% HPD: 1920–2006). Similar to Brazil, Argentina received a large number of Arab immigrants until the mid-20^th^ century, mainly originating from Syria and Lebanon [64].

Spatiotemporal reconstruction of subgenotype D2 suggested that this lineage originates in Central/Eastern Europe (Estonia, Poland, Russia, and Serbia), in agreement with previous reports [24], highlighting Russia as the most probable location of origin. The clade containing most of the Brazilian and Argentine sequences had a tMRCA around the second half of the 20^th^ century (95% HPD: 1941–1984), with Central/Eastern Europe as the most probable place of origin. Although Latin America received the majority of immigrants from Southern Europe, there was also a considerable flow of migrants from Central, East and Southeastern European countries [65]. Between 1910 and 1929, more than 1.5 million immigrants from Central and Eastern Europe (mainly Poland, Romania, Russia, Lithuania, and Latvia) entered Brazil for employment in agriculture [66]. On the other hand, India was estimated to be the most likely location for origin of subgenotype D2 in USA. Notably, HBV/D is very prevalent in India, where at least five circulating subgenotypes have been identified (D1 to D5) [67–69] and the second largest group of immigrants in USA is from India [70].

Dispersal pathways of subgenotype D3 in the Americas were complex including different geographic regions and multiple introductions. Phylogeographic analysis suggested Southern Europe (Italy and Spain) (*PSP* = 0.54) as the root location of D3, and Brazil as the origin place of most dispersal pathways occurring from the late 18th century in the continent. Previous studies based on epidemiological and historical data have suggested that subgenotype D3 was transported to Brazil by European immigrants [55, 56]. Of note, mass European emigration to the Americas took place from the early 19th to the mid-20th century, especially to the United States, Argentina, Canada, Brazil, Cuba, and Uruguay. About 13 million Europeans went to Latin America, most of them were Italians, Spaniards and Portuguese [65]. However, the lack of complete genome sequences representing all variations of European D3 may have limited our analysis, leading to indications of Brazil as the source location of most dispersal pathways in the continent.

The spatiotemporal reconstruction of subgenotype D4 suggested that Martinique is the most likely location of origin of this subgenotype. This result is incompatible with epidemiological and historical data of HBV in the Americas. In the American countries, D4 has been identified mainly in African descendant populations [31, 33, 34, 71, 72], with the exception of D4 strains found in individuals living in the Southwestern region of the Canadian Arctic [73]. Brazil and the Caribbean received a large number of African slaves between the 16^th^ and 19^th^ centuries [74, 75]. Some earlier studies suggest an African origin for subgenotype D4 [31, 71], but none to date have performed Bayesian phylogeographic analysis. Notably, D4 has been described in Rwanda [76], Somalia [77], Kenya [78] and Ghana [79]. However, only one complete African D4 genome sequence is available (GenBank accession number KF922432), which may have limited our analysis leading to estimates of Martinique as the place of origin of this subgenotype. Based on the collective evidence, our hypothesis is that the D4 sequences from Martinique represent sequences from ancestral African populations that have introduced this subgenotype in the Americas.

In conclusion, this study highlights the differential distribution patterns and evolutionary dynamics of HBV/D in the Americas, supporting the utility of recently advanced phylogenetic tools in reconstructing the evolutionary history of HBV, and provides novel full-length HBV/D genomic sequences, increasing the contribution of Brazilian isolates to ongoing phylogenetic and phylogeographic studies.

## Supporting information

S1 Table GenBank accession numbers of the 421 full-length HBV/D sequences used in Bayesian phylogeographic analyses. (DOCX)

S1 Fig Phylogenetic analysis of HBV complete genome sequences. ML phylogenetic tree of 39 Brazilian HBV/D complete genomes recovered in this study plus 27 reference sequences. The sequences generated are denoted BR, followed by sample number and the geographic region of origin (NE, North East; CW, Central West; SE, South East; S, South), and identified with the symbol ♦. Reference sequences are indicated by their accession number, followed by genotype. The numbers at nodes correspond to bootstrap values (1000 replicates) higher than 70%.
(TIF)

S2 Fig Likelihood-mapping analysis of the four HBV/D datasets. (A) HBV/D1; (B) HBV/D2; (C) HBV/D3; (D) HBV/D4; The triangles show the distribution (percentages) of the data in the seven basins of attraction or quartet possibilities. The percentages in the corners and sides represent well resolved phylogenies, while the percentage in the center of the triangle represents unresolved phylogenies.
(TIF)

S3 Fig Root-to-tip regression analyses of temporal structure. Plots of the root-to-tip genetic distance against sampling time are shown for phylogenies estimated from four alignments: (A) HBV/D1; (B) HBV/D2; (C) HBV/D3 and (D) HBV/D4. Each dot represents a sequence. R^2^ values are given as an indicator of the degree to which evolution has been clock-like.
(TIF)
