# Supplementary material for: Reconstruction of the spatial and temporal dynamics of hepatitis B virus genotype D in the Americas
Source: PLoS One. 2019 Jul 25;14(7):e0220342. doi: 10.1371/journal.pone.0220342 (PMC6657902; doi:10.1371/journal.pone.0220342)
Supplement: S1 Table — (DOCX) [file pone.0220342.s001.docx]

**Supplementary file 1.** GenBank accession numbers of the 421 full-length HBV/D sequences used in the Bayesian phylogeographic analyses.

| AB048701 | AB583679 | DQ464178 | FJ349220 | GQ477453 |
| --- | --- | --- | --- | --- |
| AB048702 | AB583680 | DQ464181 | FJ349221 | GQ477455 |
| AB048703 | AB583681 | DQ464182 | FJ349228 | GQ477456 |
| AB078031 | AJ344116 | DQ486021 | FJ349229 | GQ477457 |
| AB078032 | AJ627215 | DQ486022 | FJ349230 | GQ477458 |
| AB090268 | AJ627216 | DQ486024 | FJ349231 | GQ477459 |
| AB090269 | AJ627217 | DQ486025 | FJ349232 | GQ922000 |
| AB090270 | AJ627218 | EU594396 | FJ349233 | GQ922001 |
| AB109478 | AJ627220 | EU594397 | FJ349234 | GQ922002 |
| AB116266 | AJ627221 | EU594398 | FJ349235 | GQ922003 |
| AB119252 | AJ627222 | EU594399 | FJ386590 | GQ922004 |
| AB119253 | AJ627223 | EU594400 | FJ562309 | GQ922005 |
| AB119254 | AJ627224 | EU594404 | FJ562338 | GU456635 |
| AB119255 | AY236162 | EU594406 | FJ692506 | GU456636 |
| AB119256 | AY902768 | EU594408 | FJ692507 | GU456638 |
| AB120308 | AY902769 | EU594409 | FJ692532 | GU456677 |
| AB210820 | AY902770 | EU594415 | FJ692533 | GU456678 |
| AB210822 | AY902772 | EU594422 | FJ692536 | GU456679 |
| AB267090 | AY902773 | EU594424 | FJ899792 | GU456682 |
| AB270538 | AY902774 | EU594425 | FJ904399 | GU456684 |
| AB270539 | AY902776 | EU594426 | FJ904402 | GU563560 |
| AB270541 | AY902777 | EU594428 | FJ904412 | HE974372 |
| AB270542 | DQ304547 | EU594430 | FJ904415 | HE974373 |
| AB270543 | DQ304548 | EU594434 | FJ904420 | HE974378 |
| AB270546 | DQ304549 | EU594435 | FJ904421 | HE974379 |
| AB270547 | DQ304550 | EU921418 | FJ904424 | HE974382 |
| AB270548 | DQ304551 | EU921419 | FJ904426 | HQ700446 |
| AB270550 | DQ329357 | EU939680 | FJ904427 | HQ700458 |
| AB330366 | DQ336690 | FJ349205 | FJ904432 | HQ700459 |
| AB330367 | DQ336692 | FJ349206 | FJ904443 | HQ700463 |
| AB330368 | DQ464164 | FJ349208 | FJ904445 | HQ700472 |
| AB330369 | DQ464165 | FJ349209 | FJ904446 | HQ700478 |
| AB330370 | DQ464166 | FJ349210 | GQ183448 | HQ700480 |
| AB554016 | DQ464167 | FJ349212 | GQ183470 | HQ700481 |
| AB554023 | DQ464168 | FJ349213 | GQ183472 | HQ700488 |
| AB554024 | DQ464169 | FJ349214 | GQ183475 | HQ700492 |
| AB555496 | DQ464170 | FJ349215 | GQ183480 | HQ700493 |
| AB555497 | DQ464172 | FJ349216 | GQ183484 | HQ700494 |
| AB555500 | DQ464173 | FJ349218 | GQ183486 | HQ700497 |
| AB555501 | DQ464175 | FJ349219 | GQ477452 | HQ700500 |

| HQ700501 | JF754630 | JN642143 | JN688716 | KF192839 |
| --- | --- | --- | --- | --- |
| HQ700503 | JF754631 | JN642144 | JN688717 | KF192840 |
| HQ700510 | JF754632 | JN642146 | JQ687530 | KF192841 |
| HQ700511 | JF754633 | JN642147 | JQ687531 | KF471648 |
| HQ700512 | JF754634 | JN642148 | JQ687532 | KF584158 |
| HQ700513 | JF754635 | JN642149 | JQ707505 | KF584159 |
| HQ700514 | JN040753 | JN642150 | JQ707508 | KF584160 |
| HQ700524 | JN040762 | JN642154 | JQ707530 | KF584161 |
| HQ700525 | JN040763 | JN642157 | JQ707706 | KF584162 |
| HQ700533 | JN040769 | JN642158 | JX470760 | KF584163 |
| HQ700534 | JN040773 | JN642159 | JX898694 | KF584164 |
| HQ700535 | JN040779 | JN642160 | JX898695 | KF584165 |
| HQ700536 | JN040782 | JN642161 | JX898696 | KF584166 |
| HQ700537 | JN040818 | JN642163 | JX898697 | KJ470884 |
| HQ700538 | JN257148 | JN642167 | JX898698 | KJ470885 |
| HQ700540 | JN257149 | JN664909 | JX898699 | KJ470886 |
| HQ700541 | JN257150 | JN664910 | KC774436 | KJ470887 |
| HQ833465 | JN257152 | JN664912 | KC774437 | KJ470888 |
| HQ833467 | JN257154 | JN664916 | KC774440 | KJ470889 |
| HQ833468 | JN257160 | JN664928 | KC774444 | KJ470890 |
| HQ833469 | JN257162 | JN664930 | KC774445 | KJ470891 |
| HQ833470 | JN257165 | JN664931 | KC774450 | KJ470893 |
| HQ833471 | JN257170 | JN664936 | KC774459 | KJ470894 |
| JF754586 | JN257172 | JN664938 | KC774460 | KJ470895 |
| JF754588 | JN257174 | JN664939 | KC774462 | KJ470896 |
| JF754592 | JN257177 | JN664941 | KC875275 | KJ470897 |
| JF754594 | JN257181 | JN664942 | KC875292 | KJ470898 |
| JF754597 | JN257182 | JN664944 | KC875301 | KJ647349 |
| JF754600 | JN257183 | JN688678 | KC875312 | KJ647350 |
| JF754609 | JN257185 | JN688679 | KC875327 | KJ647351 |
| JF754611 | JN257186 | JN688683 | KC875337 | KJ647352 |
| JF754612 | JN257188 | JN688685 | KF192830 | KJ647353 |
| JF754613 | JN257189 | JN688689 | KF192831 | KJ647355 |
| JF754617 | JN257190 | JN688695 | KF192832 | KJ647356 |
| JF754619 | JN257204 | JN688708 | KF192833 | KJ843187 |
| JF754620 | JN642128 | JN688710 | KF192834 | KM359442 |
| JF754621 | JN642135 | JN688711 | KF192835 | KM386676 |
| JF754625 | JN642137 | JN688712 | KF192836 | KM577663 |
| JF754626 | JN642138 | JN688713 | KF192837 | KM577664 |
| JF754629 | JN642142 | JN688715 | KF192838 | KM577665 |

| KM577666 |  |  |  |  |
| --- | --- | --- | --- | --- |
| KM577667 |  |  |  |  |
| KM577668 |  |  |  |  |
| KM577669 |  |  |  |  |
| KM577670 |  |  |  |  |
| KM577671 |  |  |  |  |
| KM606740 |  |  |  |  |
| KM606744 |  |  |  |  |
| KM606745 |  |  |  |  |
| KM606752 |  |  |  |  |
| KM606753 |  |  |  |  |
| KM606754 |  |  |  |  |
| KM606755 |  |  |  |  |
| KP322601 |  |  |  |  |
| KP322602 |  |  |  |  |
| KT749845 |  |  |  |  |
| KT749845 |  |  |  |  |
| KT963508 |  |  |  |  |
| KU736926 |  |  |  |  |
| KU736927 |  |  |  |  |
| KX827292 |  |  |  |  |
| KX827302 |  |  |  |  |
|  |  |  |  |  |
|  |  |  |  |  |
|  |  |  |  |  |
|  |  |  |  |  |
|  |  |  |  |  |
|  |  |  |  |  |
|  |  |  |  |  |
|  |  |  |  |  |
|  |  |  |  |  |
|  |  |  |  |  |
|  |  |  |  |  |
|  |  |  |  |  |
|  |  |  |  |  |
|  |  |  |  |  |
|  |  |  |  |  |
|  |  |  |  |  |
|  |  |  |  |  |
|  |  |  |  |  |
