# Supplementary figures and images for: Reconstruction of the spatial and temporal dynamics of hepatitis B virus genotype D in the Americas
Source: PLoS One. 2019 Jul 25;14(7):e0220342. doi: 10.1371/journal.pone.0220342 (PMC6657902; doi:10.1371/journal.pone.0220342)

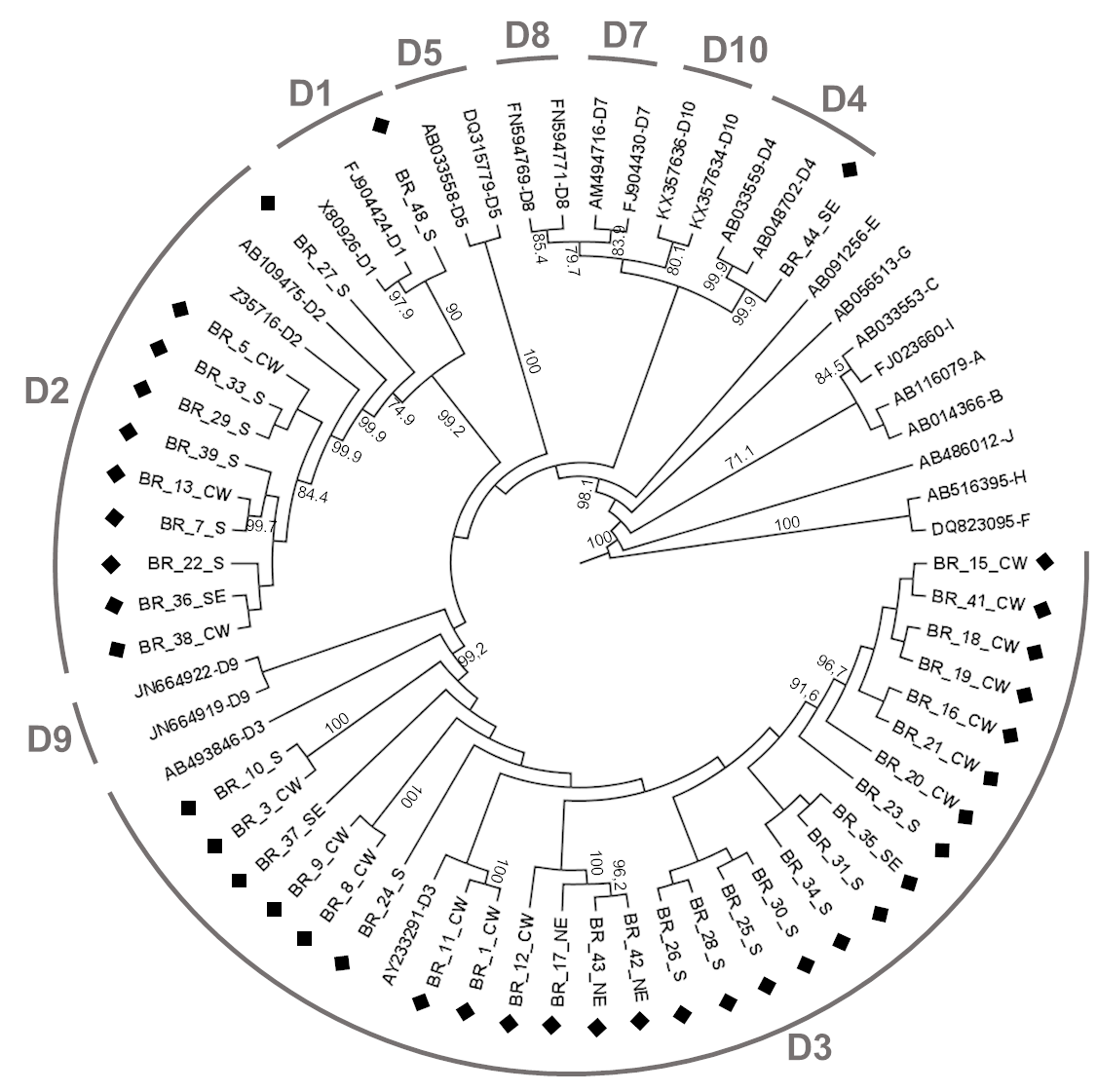

Supplement: S1 Fig — ML phylogenetic tree of 39 Brazilian HBV/D complete genomes recovered in this study plus 27 reference sequences. The sequences generated are denoted BR, followed by sample number and the geographic region of origin (NE, North East; CW, Central West; SE, South East; S, South), and identified with the symbol ♦. Reference sequences are indicated by their accession number, followed by genotype. The numbers at nodes correspond to bootstrap values (1000 replicates) higher than 70%. (TIF) [file pone.0220342.s002.tif]

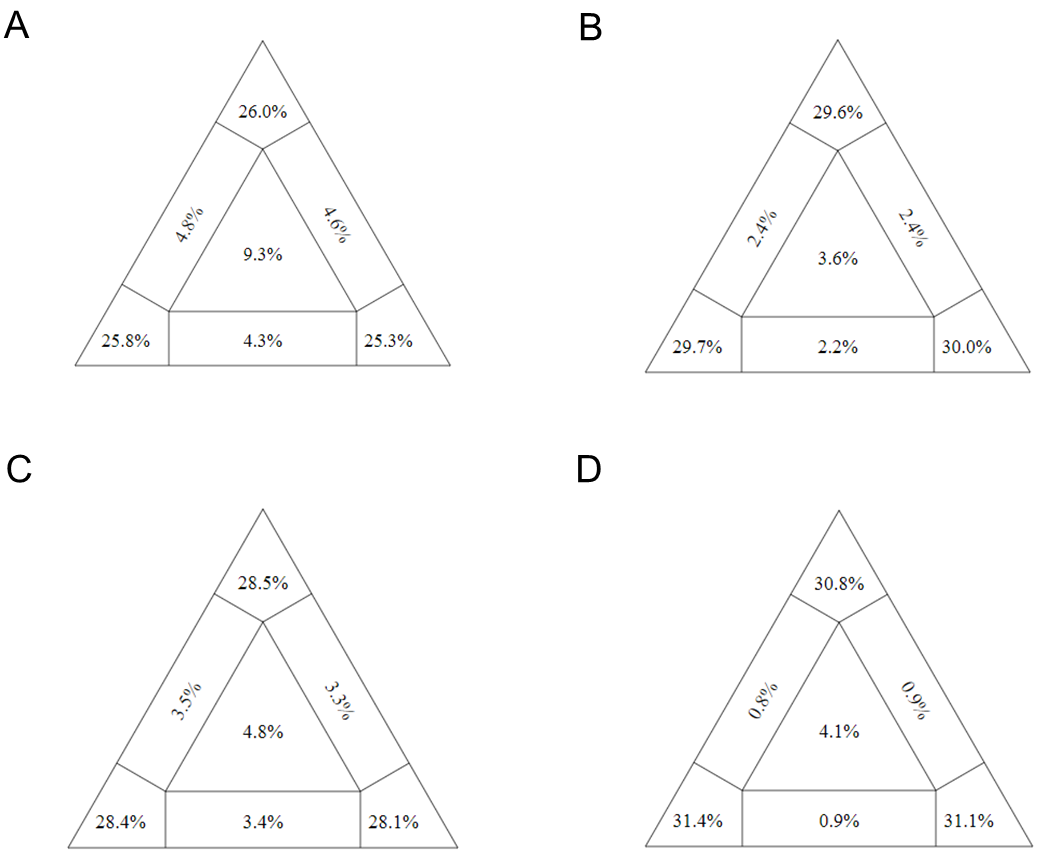

Supplement: S2 Fig — (A) HBV/D1; (B) HBV/D2; (C) HBV/D3; (D) HBV/D4; The triangles show the distribution (percentages) of the data in the seven basins of attraction or quartet possibilities. The percentages in the corners and sides represent well resolved phylogenies, while the percentage in the center of the triangle represents unresolved phylogenies. (TIF) [file pone.0220342.s003.tif]

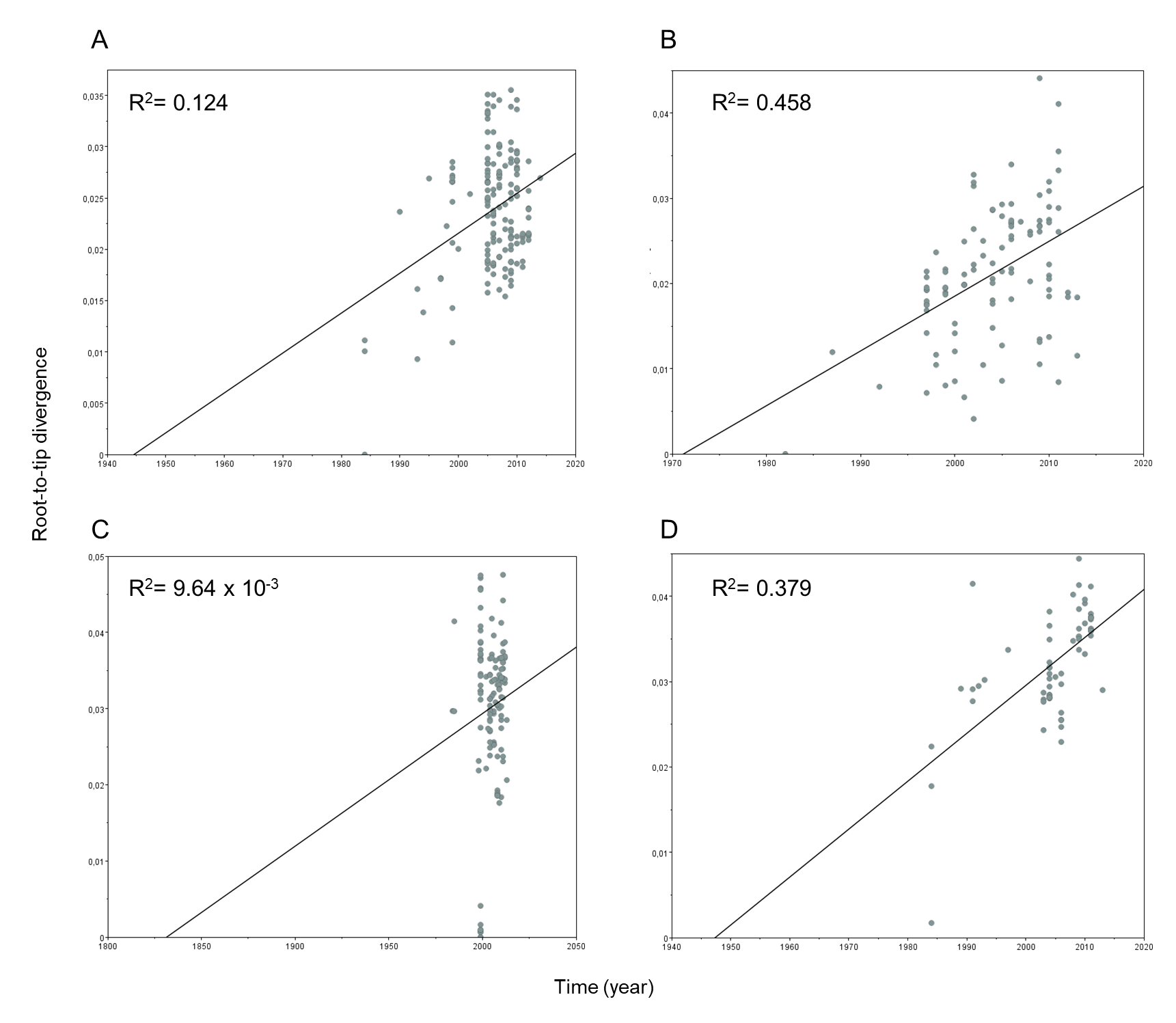

Supplement: S3 Fig — Plots of the root-to-tip genetic distance against sampling time are shown for phylogenies estimated from four alignments: (A) HBV/D1; (B) HBV/D2; (C) HBV/D3 and (D) HBV/D4. Each dot represents a sequence. R2 values are given as an indicator of the degree to which evolution has been clock-like. (TIF) [file pone.0220342.s004.tif]
